# Hydraulic system fault diagnosis decoupling method based on 2D time-series modeling and self-attention fusion

**DOI:** 10.1038/s41598-024-66541-9

**Published:** 2024-07-07

**Authors:** Haicheng Wang, Juan Zhou, Hu Chen, Bo Xu, Zhengxiang Shen

**Affiliations:** 1 College of Energy Environment and Safety Engineering and College of Carbon Metrology, China Jiliang University, Hangzhou, 310018 China; 2 Ningbo Special Equipment Inspection and Research Institute, Ningbo, 315000 China

**Keywords:** Energy infrastructure, Mechanical engineering

## Abstract

Hydraulic systems play a pivotal and extensive role in mechanics and energy. However, the performance of intelligent fault diagnosis models for multiple components is often hindered by the complexity, variability, strong hermeticity, intricate structures, and fault concealment in real-world conditions. This study proposes a new approach for hydraulic fault diagnosis that leverages 2D temporal modeling and attention mechanisms for decoupling compound faults and extracting features from multisample rate sensor data. Initially, to address the issue of oversampling in some high-frequency sensors within the dataset, variable frequency data sampling is employed during the data preprocessing stage to resample redundant data. Subsequently, two-dimensional convolution simultaneously captures both the instantaneous and long-term features of the sensor signals for the coupling signals of hydraulic system sensors. Lastly, to address the challenge of feature fusion with multisample rate sensor data, where direct merging of features through maximum or average pooling might dilute crucial information, a feature fusion and decoupling method based on a probabilistic sparse self-attention mechanism is designed, avoiding the issue of long-tail distribution in multisample rate sensor data. Experimental validation showed that the proposed model can effectively utilize samples to achieve accurate fault decoupling and classification for different components, achieving a diagnostic accuracy exceeding 97% and demonstrating robust performance in hydraulic system fault diagnosis under noise conditions.

## Introduction

Hydraulic systems play a pivotal role in modern mechanical design, boasting advantages such as rapid response characteristics, high load stiffness, remarkable power density, and commendable stability performance, leading to widespread adoption in various applications. Typically forming the core of engineering equipment, especially in control and power transmission, they are often operated in field environments. However, hydraulic systems can be vulnerable to damage due to handling high loads, experiencing severe shocks, or facing unstable working conditions. Notably, the failure of internal components within a hydraulic system can initiate a chain reaction, resulting in the cascading failure of multiple parts^1,2^. Consequently, timely fault diagnosis and troubleshooting of hydraulic systems to ensure their smooth operation and the efficiency of engineering equipment are of paramount importance.

Nevertheless, hydraulic systems exhibit a coupling characteristic encompassing mechanical, electrical, and hydraulic elements, rendering accurate fault diagnosis a formidable challenge. Compared to the more transparent failures in common electromechanical structures, malfunctions within hydraulic systems in engineering equipment are notably more obscure and ambiguous, characterized by the following aspects:

- Compared to conventional electrical and mechanical systems, hydraulic systems boast enhanced hermeticity, intricate structures, and a propensity for concealed malfunctions, rendering them susceptible to disturbances. For instance, the reciprocating motion of cylinders generates a significant amount of excitation sources^3^.
- The mapping between the characteristics of malfunctions and their underlying causes is intricate. A single fault may stem from numerous sources, making the precise identification of the origin challenging. The failure of internal components can initiate a domino effect, leading to the cascading failure of multiple parts.
- Failures within the hydraulic system can occur either concurrently or independently. When composite malfunctions arise, the signals transmitted through the intricate channels of the hydraulic system may become distorted, thereby impacting the accuracy of fault detection outcomes.

The quintessence of diagnostic methodologies for hydraulic system malfunctions lies in extracting signal characteristics and decoupling fault diagnoses, thus establishing a correlation between fault manifestations and their underlying causes. To date, many scholars have delved into the realm of fault diagnosis within hydraulic systems, employing fundamental techniques such as signal processing, statistical methodologies, machine learning, and deep learning to forge intelligent fault diagnosis methodologies.

In the initial stages, experts and technicians directly observed and analyzed the components’ failure mechanisms for subjective diagnostics or engaged in modeling and simulation^1^. For instance, they constructed simulation models using AMEsim software to analyze the fault modes and mechanisms of critical components^4^; Subsequently, scholars introduced sensor technology and signal processing techniques into hydraulic systems for feature extraction. They described the relationship between measured signals and fault modes through mathematical models, which typically encompassed traditional statistical methods and machine learning approaches^5^. This includes, but is not limited to, various signal decomposition techniques such as Continuous Wavelet Transform (CWT), Filter Bank-based Empirical Wavelet Transform (FBEWT), Ensemble Empirical Mode Decomposition (EEMD), and Wavelet Packet Decomposition Reconstruction (WPDR). Fault decoupling algorithms mainly consist of physical model correction methods based on cosine similarity^6^, Linear Discriminant Analysis (LDA)^7^, and Extreme Gradient Boosting (XGBoost)^8^.

As the discipline of fault diagnosis increasingly transitions into the vast and intricate landscape of big data, the plethora of collected signals is both immense in volume and multifaceted. Conventional methodologies for identification are finding themselves poorly equipped to cater to the exigencies presented by the tremendous influx of data. Therein lies the importance of harnessing deep learning techniques to forge intelligent diagnostic algorithms within this domain. The prowess of Convolutional Neural Networks (CNNs) in deep learning, with their inherent capability for autonomous feature learning and the advantages presented by their local receptive fields in extracting features, has been recognized with accolades in computer vision. Presently, these methodologies are being zealously adopted in fault diagnosis. For example, Tang SN, along with associates, made notable advancements by applying the continuous wavelet transformation to morph raw vibration signals from hydraulic axial piston pumps into bidimensional time-frequency representations, subsequently employing profound CNN networks for the extraction of features and the diagnosis of faults.

Similarly, Zhu and his team^9^, after transforming signals into the time-frequency domain via wavelet changes, improved and stacked the classic CNN network, AlexNet, for fault diagnosis tasks. Jiang et al.^10^, replaced ordinary convolutional kernels with stochastic ones for feature extraction from the vibration signals of axial piston pumps. Another approach involves the direct use of one-dimensional convolutional methods, the advantage of which lies in their suitability for handling sequential data. This allows for capturing local patterns and long-range dependencies within sequences while preserving the sequential order information.Such methods have extensively applied in numerous natural language processing and time series analysis tasks^11^. Huang et al.^12^, have harnessed the capabilities of multi-layer, multi-channel 1D CNNs to devise a diagnostic model for hydraulic system components, leveraging multispeed data samples collected from various sensors. In pursuit of enhancing the reliability of deep neural networks for industrial fault diagnosis and condition monitoring, Keleko AT, among others^13^, has integrated the game-theoretic approach of Shapley Additive exPlanations into neural networks. This integration aims to furnish credible outcomes by elucidating each sensor’s significant weights and contributions towards the DNN’s decision-making process, a methodology whose efficacy has been corroborated through tests on hydraulic system data.

The aforementioned deep learning methodologies are adept at adaptively extracting fault characteristics, exhibiting robust fault classification capabilities. However, applying deep learning algorithms for fault diagnosis requires a substantial volume of samples, requiring single-label samples of various fault modes. In hydraulic systems, faults tend to be covert and intricate, often manifesting as composite and coupled. Therefore, the utilization of multi-sensor feature information fusion technology is advisable.

Integrating multi-sensor feature information technology is an emerging trend in fault diagnosis. It can generate more comprehensive information than individual sensors, thereby improving the robustness of fault diagnosis and addressing the issue of complex fault decoupling^12^. The focus of current research lies in how to extract features from time series data of different sensors and fuse the features from sensors with different sampling rates. The fundamental approach to feature information fusion is to utilize multiple channels for extracting feature information, where each channel independently extracts features based on dimensions such as time and frequency. Afterward, the information from these channels is integrated.

Thus, extracting multi-channel features from sensor arrays operating at various sampling rates is a pivotal challenge in diagnosing hydraulic system malfunctions.

The employment of temporal features derived from sensor data timestamps stands as a critical solution, with time series analysis extensively applied in detecting anomalies and predicting life expectancy in industrial monitoring data^14^. Methods utilizing Recurrent Neural Networks (RNN)^15,16^, predicated on the Markov assumption for modeling continuous time, and those employing Convolutional Neural Networks (CNN) on the temporal dimension to capture changes^17,18^, underscore the fundamental task of time series feature extraction: to distill both long-term characteristics and short-term features, mirroring the temporal data of industrial sensors in capturing the immediate variability and enduring trends of sensor data^19,20^.

Another critical aspect of diagnosing hydraulic system failures is the fusion of features from sensors operating at multiple sampling rates. Despite the formidable feature extraction capabilities of CNNs, critical information may be diminished when features within the model are directly amalgamated using maximum or average pooling.

Attention mechanisms have effectively solved this problem. For example, Song et al.^21^ constructed a new deep transfer learning network model for planetary gearbox fault diagnosis by integrating one-dimensional convolutional neural networks, attention mechanisms, and domain adaptation methods; Zhao et al.^22^ utilized attention mechanisms to share information between channels, and their constructed rotor system fault diagnosis model demonstrated excellent load adaptability and noise resistance; Cheng et al.^23^ designed a lightweight and efficient channel attention mechanism (LECA) combined with one-dimensional convolution, achieving good gearbox fault diagnosis results; Zhang et al.^24^ applied attention mechanisms in unsupervised fault diagnosis, developing a semi-supervised attention mechanism to solve the problems of insufficient fault diagnosis data and false alarms in online fault diagnosis.

This study proposes a hydraulic system fault diagnosis method based on temporal two-dimensional variation modeling and the fusion of multi-rate data samples. This method addresses the issues above, facilitating the decoupled diagnosis of fault-coupled hydraulic systems in critical components. The primary contributions of this paper are as follows:

1. To mitigate the issue of oversampling in high-frequency sensors, a variable frequency data sampling method is introduced to streamline redundant high-frequency data.
2. In response to the strong concealment and high coupling of faults in hydraulic systems, a two-dimensional change modeling of sensor temporal data is employed, utilizing a two-dimensional convolution method to extract both the instantaneous variability and long-term trends of sensor data, overcoming the limitations of one-dimensional feature extraction methods.
3. The proposed feature fusion framework, based on the attention mechanism, automatically integrates features from multi-rate data samples, superseding the traditional approach of merging features within CNN networks through direct maximum or average pooling, thereby ameliorating the issue of crucial information loss.

## Methods

**Figure 1:**
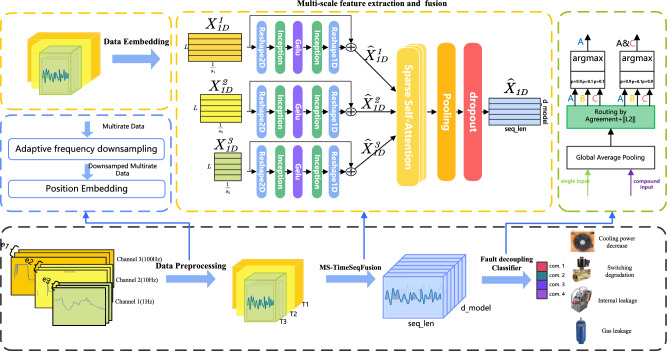
Fault diagnosis process of multi-scale time series decoupling.

The process of diagnosing faults in multi-scale time series involves three stages, as illustrated in Fig. 1: Multi-rate data preprocessing, multi-scale time series feature extraction and fusion, and faulty decoupling and classification. During data preprocessing, high-frequency signals are resampled, and all signals are encoded with their sequence positions. For multi-scale feature extraction and fusion, sensor data with different sampling rates are transformed into 2D representations per channel. Convolutions are then employed to extract short-term and long-term period features from a 2D perspective. Feature fusion uses self-attention to focus on deep features and feature decoupling at various scales.

### Multi-rate data preprocessing

**Figure 2:**
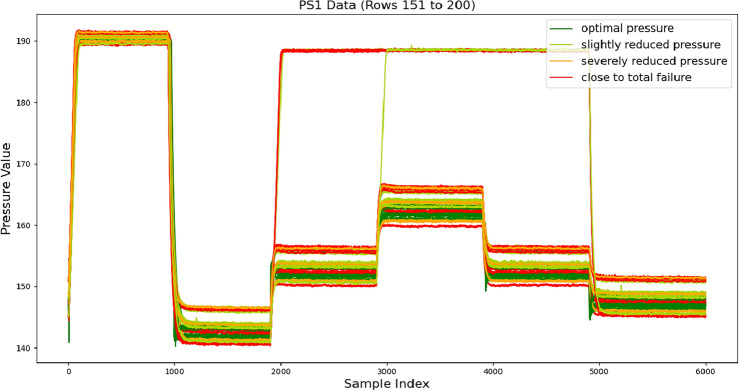
The first 50 sets of data from pressure sensor PS1.

The extraction and fusion of data features from multiple sensors have always been topics of intense research. Different sensors measure various physical quantities, and their data features vary in scale. These data feature mappings provide insights into the conditions of various components. By utilizing data fusion methods, the accuracy of fault diagnosis can be enhanced. However, due to the multi-rate characteristics of measurement data and the different sampling frequencies of multiple sensors, it is impossible to process the input data in the same dimension. Therefore, it is necessary to preprocess the data. Upsampling and downsampling methods are not suitable for the current application scenario. For example, downsampling involves sampling in high-frequency data, and the detection performance of accumulator component faults relies on instantaneous variability.

Figure 2 demonstrates the data of pressure sensor PS1. It has been observed that high-frequency sensors, including pressure sensors, exhibit significant changes in data frequency and amplitude when malfunctioning in the hydraulic system. This type of data characteristic is common in mechanical systems, and if high-frequency data is downsampled, the information on these data changes may be lost. Therefore, analyzing time series data aims to capture the instantaneous changes during signal transients and the long-term trends of signal trends. It is generally difficult and ineffective to extract two features on a one-dimensional time series simultaneously. The sampling frequency of the pressure sensor is 100 Hz, which results in oversampling due to the relatively lower frequency of pipeline pressure changes. Additionally, the hydraulic platform simulation in this dataset encompasses testing variable work cycles with pseudo-random load variations and fixed work cycles with predetermined load levels. These scenarios represent typical cyclic operations and repetitive load characteristics found in industrial applications, as well as load fluctuations in mobile machinery that undergo local abrupt changes during operation. High-frequency sensors within hydraulic systems will exhibit significant alterations in both frequency and amplitude upon failure. This type of data feature is prevalent in mechanical systems; direct downsampling of high-frequency data may lead to a loss of crucial information regarding these data changes. Therefore, it is necessary to conduct variable-frequency sampling on high-frequency data and analyze time series data to capture instantaneous signal changes during transient conditions and long-term trends.

Industrial sensors include those with high sampling frequencies, such as pressure sensors for hydraulic systems, which have a sampling frequency of 100 Hz. However, the pressure variation in the hydraulic system does not reach this frequency, resulting in oversampled data. Traditional uniform downsampling methods have issues. Setting the sampling frequency too high can capture complete data information, but if there are many sensors and components, it generates a large amount of data, reducing the efficiency of fault diagnosis models. On the other hand, setting the sampling frequency too low requires more data to guarantee the accuracy of data collection, which may lead to the loss of valuable data, hindering the diagnosis of faults in hydraulic coupling systems under complex conditions.

The problem can be solved using variable-frequency data sampling. Variable-frequency data sampling has been used in data collection, where sensors analyze the variations in previously collected data and automatically adjust the time interval for data sampling. In the data processing stage, this method is used to downsample the collected data. In simple terms, variable-frequency data sampling adjusts the sampling frequency based on data changes.

This paper utilizes an integrated variable-frequency downsampling method based on the jitter ratio and swing door algorithm^25^. Firstly, the basic principle of the jitter ratio method involves fitting n sampling points into a straight line using least squares, and the distance between the data points and the line represents the fluctuation level of the data. The jitter ratio for n sampling points is defined as the ratio of the average jitter size between the closest m sampling points within a data window to the remaining m − n data points, denoted as $\delta$.

1 $$\begin{aligned} \begin{array}{c} \delta =\frac{\frac{1}{m}\sum _{k=i-1}^{i-m}{|}\beta _0+\beta _1t_k-y_k|\sin \theta }{\frac{1}{n-m}\sum _{k=i-m-1}^{i-n}{|}\beta _0+\beta _1t_k-y_k|\sin \theta } \\ =\frac{(n-m)\sum _{k=i-1}^{i-m}{|}\beta _0+\beta _1t_k-y_k|}{m\sum _{k=i-m-1}^{i-n}{|\beta _0}+\beta _1t_k-y_k|} \end{array} \end{aligned}$$

The rotating gate algorithm constructs an expanding quadrilateral to store data outside the quadrilateral. It determines whether the data is inside the quadrilateral by comparing the slopes of the upper and lower rotating gates. If the upper slope is less than the lower slope, it means that the data is within the rotating gate, indicating a smooth data change. The frequency acquisition method based on the jitter ratio is more suitable for situations where the data change is relatively smooth, while the acquisition method based on the rotating gate algorithm is suitable for data acquisition processes with large data fluctuation.

To judge the severity of parameter changes using the jitter ratio, first collect historical data of the process at a relatively high frequency. Calculate the jitter ratio for each sampling point within an interval. Within a sequence of *k* consecutive jitter ratios, if the number of sampling points where $\delta$ exceeds the threshold is *h*, and if *h* is greater than *k*/2, then the parameter is considered to have undergone significant changes during the production process; otherwise, the parameter is considered to have changed smoothly. For parameters with smooth changes, data collection is conducted using a method based on the jitter ratio, with slightly longer initial data collection intervals. For parameters with significant changes, data collection is conducted using a method based on the rotating gate algorithm, with slightly shorter initial data collection intervals. The formula is expressed as follows:

2 $$\begin{aligned} \begin{array}{c} T_{next}=\left\{ \begin{array}{c} \begin{array}{cc} \begin{array}{c} \begin{array}{cc} \min \left( T+T_{inc} \right) &{},\delta <\delta _1\\ \end{array}\\ \begin{array}{cc} \min \left( T-T_{dec} \right) &{},\delta>\delta _2\\ \end{array}\\ \begin{array}{cc} T&{},\delta _1\leqslant \delta \leqslant \delta _2\\ \end{array}\\ \end{array}&{},if\,\,h>k/2\\ \end{array}\\ \begin{array}{cc} \begin{array}{c} \min \left( T+T_{inc,}T_{max} \right) \\ \max \left( T/2,T_{min} \right) \\ \end{array}&{},if\,\,h\leqslant k/2\\ \end{array}\\ \end{array} \right. \end{array} \end{aligned}$$

In the formula, *T* is the initial sampling interval, $T_{inc}$ and $T_{dec}$ are the time interval step sizes for increase and decrease in jitter-based variable frequency sampling method, $T_{max}$ and $T_{min}$ are the upper and lower limits of sampling interval for the gate-based variable frequency sampling method.

When variable time sampling is used, it can cause a problem where the lengths of each time series are not consistent. In order to establish multi-scale information fusion, it is important to pad the time series lengths so that they match the original length of the sensor data.

A sine and cosine positional encoding technique is used to capture the temporal information in time series data. The values of sine and cosine are derived from the time intervals recorded with the variable frequency sampling mentioned previously. For an input observation sequence of a sensor $\vec {o}_s=\left( o_{1},o_{2},\ldots ,o_{T} \right) , o_{i}\in {\mathbb {R}} ^f$, it is mapped to a high-dimensional space as $\vec {V}=\left( v_{1},v_{2},\ldots ,v_{T} \right) , v_{i}\in {\mathbb {R}} ^d$. For the input sequence position vector $p=\left( 0,\ldots , i,\cdots , T \right)$, where *i* is the position index of each sample in the sequence, it is mapped to the same high-dimensional space as $\vec {P}=\left( p_{1},p_{2},\ldots ,p_{T} \right) , p_{i}\in {\mathbb {R}} ^d$. The final output of an embedding layer for a scale is.

3 $$\begin{aligned} \begin{array}{c} X_e=V_e+P_e \end{array} \end{aligned}$$

### Multi-scale time series feature extraction and fusion methods

**Figure 3:**
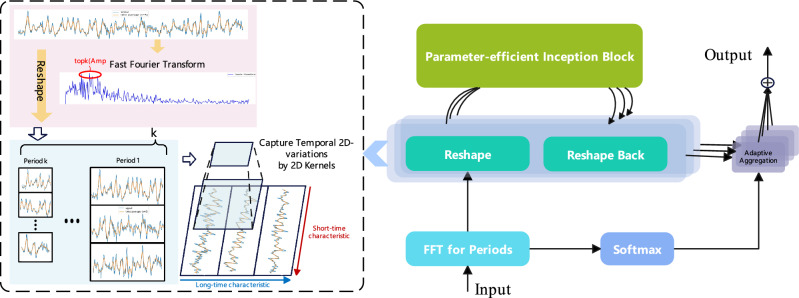
2D feature extraction methods.

Because a one-dimensional time series structure can only present changes between adjacent time points, frequency features of time series data are extracted through signal processing methods, and the time series is transformed into a two-dimensional space based on these frequency features. This explicit representation can reveal the long-term characteristics of trends and capture the short-term characteristics during transient periods, which is beneficial for subsequent representation learning. For each frequency, the 1D time series is transformed into a 2D space, and the Inception Block module with two-dimensional convolution is used to extract features for both types of changes simultaneously. As shown in Fig. 3, the top-k periods obtained from the fast Fourier transform are the data’s characteristic periods, and the two-dimensional convolution can extract both the short-term characteristics within each period and the long-term characteristics between periods. In the frequency domain, the Fast Fourier Transform (FFT) decomposes the frequencies of superimposed waves. In the resulting spectrum, frequencies with higher amplitudes are more significant.

The Pan, X’s research^26^ has shown that convolution is advantageous for extracting shallow features, while attention mechanisms are more effective for deep features. Based on this, this paper performs convolution to extract features at various scales and then uses a self-attention mechanism for feature fusion.

The comprehensive methodology for multi-scale time series feature extraction and fusion is depicted in Fig. 1. The specific process of time series feature extraction is presented in Fig. 3. Detailed outputs and data transformations at each stage are illustrated in the Supplementary Material, the specific methods include the following steps.

#### Method to convert a one-dimensional time series to two-dimensional

Time series data encompasses both short-term fluctuations and long-term trends. For instance, in a hydraulic system, there can be randomly variable working loads as well as fixed working loads. During normal operations, the hydraulic system exhibits stable long-term load changes, whereas different operational environments or fault conditions introduce random short-term load variations. Modeling these two types of features using one-dimensional time series data is challenging because the original one-dimensional structure only captures changes between adjacent time points. To address this, the time series is first transformed from the time domain to the frequency domain to capture its periodicity. This frequency domain information is then used to create a 2D representation.

For a given time series with a length of $T$ and $C$ dimensions, the initial one-dimensional representation is $X_{1D} = (\vec {x}_1, \vec {x}_2, \ldots , \vec {x}_C)$. Assuming the input data consists of C channels, each with a sampling rate of s, and each channel contains T sensors, with the top-k parameter set to k.

For the *j*-th channel, there are $T_j$ sensors. The input time series data for this channel is represented as $\left( X_{1D} \right) _j=\left( X_{1D}^{1},X_{1D}^{2},\ldots ,X_{1D}^{t},\ldots , X_{1D}^{T_j} \right)$. To represent the multi-periodic changes, it is necessary to first capture the different frequencies of the time series. This can be achieved by analyzing the time series in the frequency domain using the Fast Fourier Transform (FFT), as shown below:

4 $$\begin{aligned} X_{1D}^{l}= & {} Embedding\left( X_{1D}^{t} \right) \nonumber \\ A= & {} Mean\left( Amp\left( FFT\left( X_{1D}^{l} \right) \right) \right) , \nonumber \\ \left\{ f_1, \ldots ,f_k \right\}= & {} Topk\left( A \right) , p_{\textrm{i}}=\left[ \frac{T}{f_{\textrm{i}}} \right] , \textrm{i}\in \left\{ 1,\ldots ,k \right\} \end{aligned}$$

In the equation, $X_{1D}^t$ represents the raw data from the sensor, mapped to the input one-dimensional time series data. Embedding(.) denotes a linear layer mapping, Amp(.) represents the calculation of amplitude. A represents the amplitude for each frequency, which is averaged over the first dimension using Mean(.). Top-k represents selecting the *k* frequencies with the largest amplitudes from A, corresponding to a period of *p*. Considering the sparsity in the frequency domain and the common occurrence of noise in industrial systems, only the first *k* amplitude values are chosen, and the highest effective frequencies are obtained, denoted as $\left\{ A_{f_1},A_{f_2},\ldots ,A_{f_k} \right\}$ and $\left\{ f_1,f_2,\ldots ,f_k \right\}$, respectively, where *k* is a hyper-parameter.

Based on the selected frequencies $\left\{ f_1,f_2,\ldots ,f_k \right\}$ and their corresponding periods $\left\{ p_1,p_2,\ldots ,p_k \right\}$, the 1D time series $X_{1D}^{l}$ can be transformed into multiple 2D tensors $X_{2D}^{l,i}$ using the following equation:

5 $$\begin{aligned} \begin{array}{c} X_{2D}^{l,i}=\textrm{Reshape}_{p_i,f_i}\left( \textrm{Padding}\left( X_{1D}^{l} \right) \right) ,i\in \left\{ 1,\ldots ,k \right\} \end{array} \end{aligned}$$

Padding is to extend the time series along the time dimension with zeros in order to match its period $p_i$ with the reshaped 2D tensor, where $p_i$ and $f_i$ represent the number of rows and columns of the transformed 2D tensor, respectively. $X_{2D}^{l,i}$ represents the 2D features of the *i*-th time series based on frequency $f_i$, with its columns and rows representing changes within the period of length $p_i$ and changes between periods, respectively. Ultimately, a set of 2D tensors $\{X_{2D}^{l,1},\dots ,X_{2D}^{l,k}\}$ are obtained based on the selected frequency and estimated periods, representing k different temporal changes derived from different periods $p_i$.

The reshaping process introduces two categories of data to the transformed 2D tensor: the interplay of instantaneous variations (columns, changes occurring within a specific timeframe) and long-term features (rows, changes spanning across timeframes). Consequently, the spatial fluctuations of time-dependent modifications can be effectively handled by 2D kernels.

#### Parameter-efficient inception block

Sequence information representation can be easily obtained by using parameter-efficient Inception blocks, this method reduces the number of parameters required for 2D convolution in each channel, defined as

6 $$\begin{aligned} \begin{array}{c} {\hat{X}}_{2D}^{l,i}=\textrm{Inception}\left( \textrm{X}_{2D}^{l,i} \right) \end{array} \end{aligned}$$

This approach comes from the visual task mechanism^27^, as the time series data has been transformed into 2D, making it possible to utilize feature extraction methods from the visual domain. Here, Trunc(.) is used to restore the sequence ${\hat{X}}_{2D}^{l,i}$ to its original 1D dimension:

7 $$\begin{aligned} \begin{array}{c} {\hat{X}}_{1D}^{l}=\textrm{Trunc}\left( \textrm{Reshape}_{1,\left( p_i\times f_i \right) }\left( {\hat{X}}_{2D}^{l,i} \right) \right) , i\in \left\{ 1,\ldots ,k \right\} \end{array} \end{aligned}$$

#### Multi-scale feature fusion and decoupling

The above method achieves feature extraction for time-series data with varying rates and sampling frequencies, addressing the issue of input data that cannot be processed in the same dimension.A modified Time Block is employed to develop a multi-scale feature extraction method, with hyperparameters specifically designed for high-frequency and low-frequency data, respectively.

As shown in Fig. 1, in order to effectively utilize temporal information, the data is input into the multi-scale network after being encoded with sine and cosine position encoding. After feature extraction, each channel will have an output sequence denoted as $X_{1D}^l(e)$.

Please refer to Tables A1–A3 in the Appendix. Through correlation analysis, it is discovered that hydraulic data contains interrelated redundant information. By leveraging the advantages of the self-attention mechanism, the diagnostic model automatically integrates the features from different channels. The traditional self-attention mechanism is characterized by high time and space complexity. Studies have found that the attention weight distributions for different query-value pairs are not all highly focused, with some approaching a uniform distribution, which is referred to as lazy distribution, while the parts with higher focus are called active distribution. To improve computational efficiency, a probabilistic sparse self-attention mechanism is adopted^28^.

The standard self-attention mechanism is defined based on tuple inputs (i.e., query, key, and value). It performs scaled dot-product operation $A\left( Q,K,V \right) =Softmax\left( QK^T/\sqrt{d} \right) V$, where $Q\in {\mathbb {R}} ^{L_Q\times d},K\in {\mathbb {R}} ^{L_K\times d},V\in {\mathbb {R}} ^{L_V\times d}$, and *d* refers to the input dimension. To further discuss the self-attention mechanism, let $q_i,k_i,v_i$ respectively represent the *i*-th row of *Q*, *K*, *V*. The attention for the *i*-th query is defined in the form of probability:

8 $$\begin{aligned} \begin{array}{c} A\left( q_i,K,V \right) =\sum _j^s{{\frac{k\left( q_i,k_j \right) }{\sum _l^s{k\left( q_i,k_l \right) }}}}v_j=E_{p\left( k_j|q_i \right) }\left[ v_j \right] \end{array} \end{aligned}$$

From Eq. 8, the attention value of the *i*-th query to all keys is defined as the probability $p(k_j|q_i)$, and the output is the dot product with its corresponding value *v*. The dominant dot product shifts the attention probability distribution of the corresponding query away from a uniform distribution. The “similarity” between distributions *p* and *q* can be used to distinguish important or unimportant queries. Through the Kullback-Leibler divergence $KL\left( q||p \right) =\ln \sum _{l=1}^{L_K}{\textrm{e}^{q_ik_{l}^{\textrm{T}}/\sqrt{\textrm{d}}}}-\frac{1}{L_K}\sum _{l=1}^{L_K}{q_ik_{l}^{\textrm{T}}/\sqrt{\textrm{d}}}-\ln L_K$. Removing constants, the sparsity measure of the *i*-th query is defined as

9 $$\begin{aligned} \begin{array}{c} \textrm{M}\left( q_i,\textrm{K} \right) =\ln \sum _{l=1}^{L_K}{\textrm{e}^{\frac{q_ik_{l}^{\textrm{T}}}{\sqrt{\textrm{d}}}}}-\frac{1}{L_K}\sum _{l=1}^{L_K}{\frac{q_ik_{l}^{\textrm{T}}}{\sqrt{\textrm{d}}}} \end{array} \end{aligned}$$

The first term is the Log Sum Exponential (LSE) of all keys $q_i$, and the second term is their arithmetic mean. If the $\textrm{M}\left( q_i,\textrm{K} \right)$ of the *i*-th query is greater, the probability *p* of its attention is more “diverse”, and there is a high chance of including dominant dot products in the head fields of the long-tailed self-attention distribution.

Based on the proposed measurement, probability-sparse self-attention can be achieved by allowing each key to only attend to *u* dominant queries.

10 $$\begin{aligned} \begin{array}{c} A\left( Q,K,V \right) =Softmax\left( \frac{{\overline{Q}}K^T}{\sqrt{d}} \right) V \end{array} \end{aligned}$$

where ${\overline{Q}}$ is a sparse matrix with the same size as *q*, and it only contains the top *u* scores within the sparsity measure *M*(*q*, *K*). We control the value of *u* by a constant sampling factor *c*, setting $u=c \cdot \ln L_Q$. This ensures that ProbSparse self-attention only requires computing dot products between ${\overline{Q}}$ and *K* with a complexity of ${\mathcal {O}} \left( \ln L_Q \right)$.

From a multi-head perspective, attention generates different sparse query-key pairs for each head, focusing on different features and their representations in different subspaces.In other words, the output of multi-head attention is the decoupled characteristics of multi-component composite faults.

### Fault decoupling classification method

To decouple the multi-head output features of self-attention into different single-fault classifications, a multi-classification head is needed.

First, for the matrix $y=\left[ y_1, y_2, \dots , y_{K_l} \right] ^T\in {\mathbb {R}} ^{K_l\times R_l}$, where $y_i\in {\mathbb {R}} ^{R_l\times 1}$ represents the feature (vector) at time point *i* outputted by self-attention, and $K_l$ is the total length of the feature sequence.

Then, Eq. 11 utilizes global average pooling (*gap*) and *reshape* to transform the output matrix *y* into a one-dimensional vector. This vector is then mapped to the output layer using a fully connected layer (*projection*), resulting in the final prediction vector $y_{pred}=\left[ y^1,y^2,\dots ,y^H \right] \in {\mathbb {R}} ^{H\times 1}$. The magnitude of each component $y^i$ in $y_{pred}$ represents the probability of the input sample belonging to the *i*th class. When the magnitude of $y^i$ is close to 1, the probability of the predicted class is higher.

11 $$\begin{aligned} \begin{array}{c} y_{pred}=projection\left( reshape\left( gap\left( y \right) \right) \right) \end{array} \end{aligned}$$

Finally, as shown in Eq. 12, a threshold value $\varphi$ is chosen to limit the number of predicted labels for classification. If $y^i$ is greater than the selected threshold, the classifier outputs a label of 1 for the *i*th class. For reliable classification results, good predictions should have $y_i$ approaching 1 for the corresponding actual class and approaching 0 for other classes.

12 $$\begin{aligned} \begin{array}{c} y_{output}^{i}={\left\{ \begin{array}{ll} 1, y_{pred}^{i}\geqslant \varphi \\ 0, y_{pred}^{i}<\varphi \\ \end{array}\right. } \end{array} \end{aligned}$$

**Figure 4:**
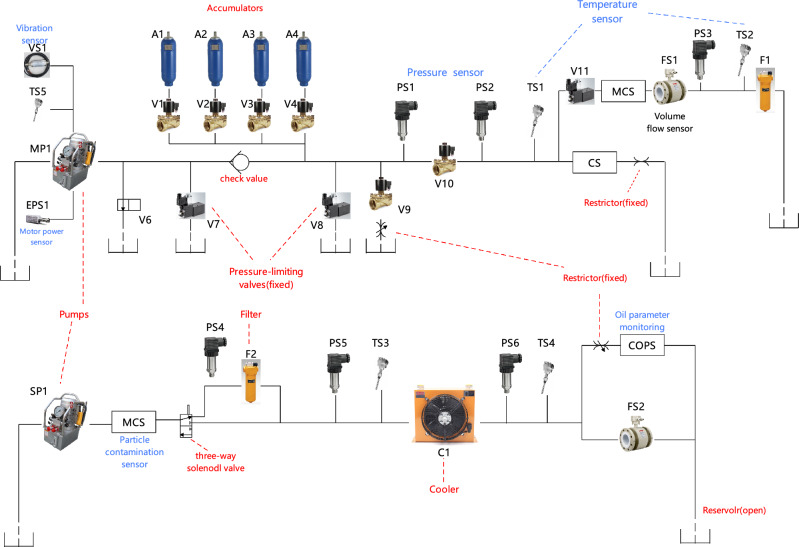
Structure of hydraulic system: primary working circuit and secondary cooling-filtration circuit.

## Experimental verification

### Dataset introduction

To validate the effectiveness of the designed model, several experiments will be conducted in this section. The fault diagnosis experimental data is sourced from an open dataset^7^.

The hydraulic engineering test bench, as shown in Fig. 4, consists of two hydraulic circuits connected through an oil tank: the primary working circuit and the secondary cooling and filtering circuit. The red-labeled components in the figure represent the critical components of the hydraulic system, while the blue labels represent sensors. The primary working circuit is the upper part of the oil circuit in Fig. 4, mainly composed of the leading pump MP1, switchable accumulators A1–A4, and filter F1. The proportional pressure relief valve (V11) generates different load levels in the main pump working circuit to simulate different conditions. The auxiliary cooling and filtering circuit is responsible for cooling and filtering the hydraulic oil, composed of a hydraulic pump SP1, three-way solenoid valve, filter F2, cooler C1, and various sensors. The micro-particle metal contamination sensor (MCS) detects the degree of hydraulic oil pollution. It determines the position detection results of the solenoid valve spool, selecting the appropriate detection method. In the figure, the solenoid valve spool is connected to the first interface, and the hydraulic oil flows through the filter to reach the condenser. If the solenoid valve slide valve is connected to another interface, the hydraulic oil will flow to the condenser without filtration.

Several types of faults can occur in the hydraulic system, with different fault types, severity, and duration. In cooler C1, the degradation level of cooling power (CP) is simulated by changing the cooling fan duty cycle. In valve V10, different switching degradation states are simulated by changing the valve current setpoint. The internal leakage level of the main pump MP1 is caused by three cascaded 0.2 mm and 0.25 mm orifices. Different leakage levels are simulated by changing the pre-charge pressure step A1–A4. The breakdown performance of different components varies. Fault characteristics such as CP reduction and valve switching degradation are obvious, making the diagnosis of these components relatively easy. However, the fault characteristics of the hydraulic pump and accumulator are more covert and easily influenced by environmental noise. Therefore, it is necessary to adopt advanced diagnostic methods to identify the faults of these components. In addition, the samples are collected under different conditions. Some data samples are taken under stable conditions (1449 samples), while others are recorded when static conditions may not yet have been reached (756 samples). The experiment uses data from these two situations, which may result in class imbalance and increase the difficulty of fault detection.

**Table 1:** Monitored parameters of the hydraulic system.

	Sensor	Physical quantity	Unit	Sampling rate
Physical sensors	PS1–PS6	Pressure	Bar	100 Hz
	FS1–FS2	Volume flow	l/min	10 Hz
	TS1–TS4	Temperature	${}^{\circ }{{\textrm{C}}}$	1 Hz
	EPS1	Motor power	W	100 Hz
	VS1	Vibration	mm/s	1 Hz
Virtual sensors	CE	Cooling efficiency	%	1 Hz
	CP	Cooling power	kW	1 Hz
	SE	Efficiency factor	%	1 Hz

**Table 2:** List of model parameter settings.

Hyper-parameters	Setting values
batch_size	32
top_k	3
learning_rate	0.001
train_epochs	30
loss	MSE
Multi-scale	3
seq_len_list	60/600/6000
e_layers	3
d_layers	1
d_model	64
d_ff	64
d_pooling	8
d_ouput	3/4

Multiple sensors are used to monitor the status of the hydraulic system. As summarized in Table 1, the physical sensors include pressure sensors (PS1–PS6), electric power sensor (EPS1), flow sensors (FS1, FS2), temperature sensors (TS1–TS5), and vibration sensor (VS1). In addition, multiple virtual sensors are utilized to provide references for the system, such as cooling efficiency (CE), CP, and system efficiency factor (SE). The data is collected on a programmable logic controller (PLC) and then transmitted to the computer through EtherCAT. The sampling rate of these sensors varies according to the dynamics of the physical quantities.

To validate the effectiveness of the model, several experiments will be conducted. Firstly, fault diagnosis of four components will be performed: cooler C1, hydraulic valve V10, main hydraulic pump MP1, and accumulators A1–A4. This will test the model’s ability to decouple single-component fault patterns from complex nested data. An exemplary fault diagnosis algorithm should be able to identify whether a component has a fault and determine the category and severity of the fault. Secondly, as the dataset is collected in a laboratory setting, it represents relatively ideal data without the complex interference of actual industrial conditions. Therefore, experiments in a low signal-to-noise ratio environment will be designed, incorporating Gaussian white noise of different decibels to test the model’s noise resistance.

In the single-component fault diagnosis experiments, X represents the data samples with multiple sampling rates, and Y represents the labels. One-hot encoding is used to diagnose a single fault in different components. Specifically,

For the faults in cooler C1:

$$\begin{aligned} Y \in \{ \text {Full efficiency}, \text {Efficiency reduction}, \text {Close to complete failure} \} = \begin{bmatrix} 1 &{} 0 &{} 0 \\ 0 &{} 1 &{} 0 \\ 0 &{} 0 &{} 1 \end{bmatrix} \end{aligned}$$

For hydraulic valve V10:

$$\begin{aligned} Y \in \{ \text {Optimal switching behavior}, \text {Slight lag}, \text {Severe lag}, \text {Close to complete failure} \} = \begin{bmatrix} 1 &{} 0 &{} 0 &{} 0 \\ 0 &{} 1 &{} 0 &{} 0 \\ 0 &{} 0 &{} 1 &{} 0 \\ 0 &{} 0 &{} 0 &{} 1 \end{bmatrix} \end{aligned}$$

For main hydraulic pump MP1:

$$\begin{aligned} Y \in \{ \text {No leakage}, \text {Weak leakage}, \text {Severe leakage} \} = \begin{bmatrix} 1 &{} 0 &{} 0 \\ 0 &{} 1 &{} 0 \\ 0 &{} 0 &{} 1 \end{bmatrix} \end{aligned}$$

For accumulators A1–A4:

$$\begin{aligned} Y \in \{ \text {Optimal pressure}, \text {Mild pressure reduction}, \text {Severe pressure reduction}, \text {Close to complete failure} \} = \begin{bmatrix} 1 &{} 0 &{} 0 &{} 0 \\ 0 &{} 1 &{} 0 &{} 0 \\ 0 &{} 0 &{} 1 &{} 0 \\ 0 &{} 0 &{} 0 &{} 1 \end{bmatrix} \end{aligned}$$

All labels of the training dataset for single-component fault diagnosis are single labels, which can be used as supervised indicators to calculate the marginal loss and supervise the model’s learning of distinguishing features. In the given hydraulic system dataset, there are 566 samples with only one type of component fault occurring, 688 samples with two types of component faults occurring simultaneously, 612 samples with three types, and 360 samples with all four components having faults.

Table 2 summarizes the hyper-parameters used in the proposed method for this experiment, including batch_size, top_k frequencies selected post FFT application, learning_rate, train_epochs, loss function, multi-scale channels “Multi-scale”, seq_len_list parameter indicating various time series data lengths in the dataset, e_layers and d_layers specifying encoder and decoder layers within the attention mechanism respectively. Additionally, it includes parameters such as d_model representing input dimension to the attention mechanism from multi-scale feature extraction output feature dimension.

**Figure 5:**
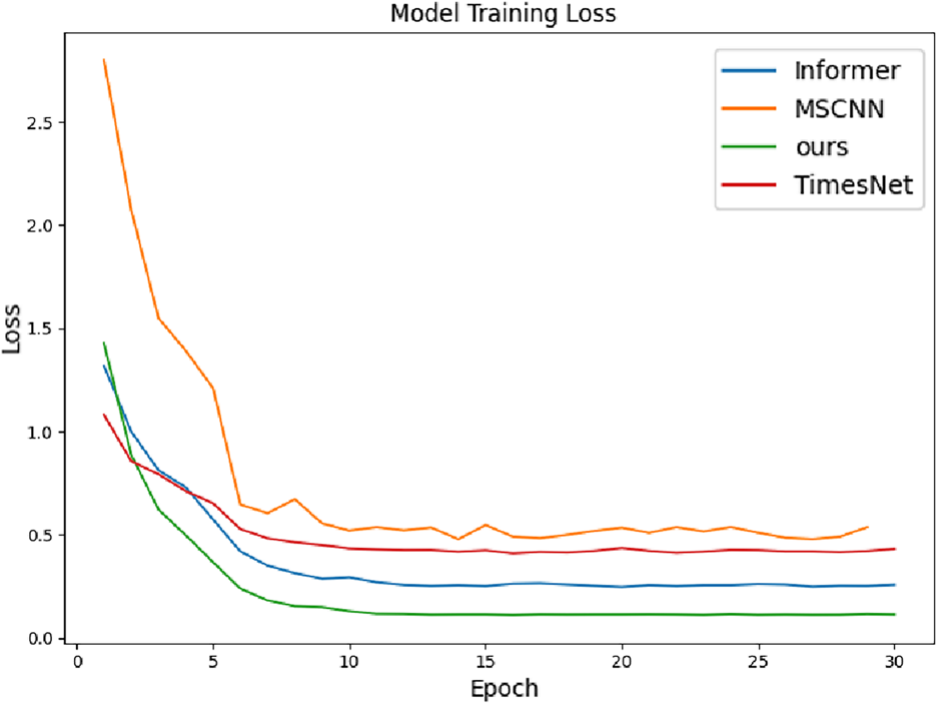
Loss changes in 30 iterations of four fault diagnosis methods in accumulator component diagnosis.

**Figure 6:**
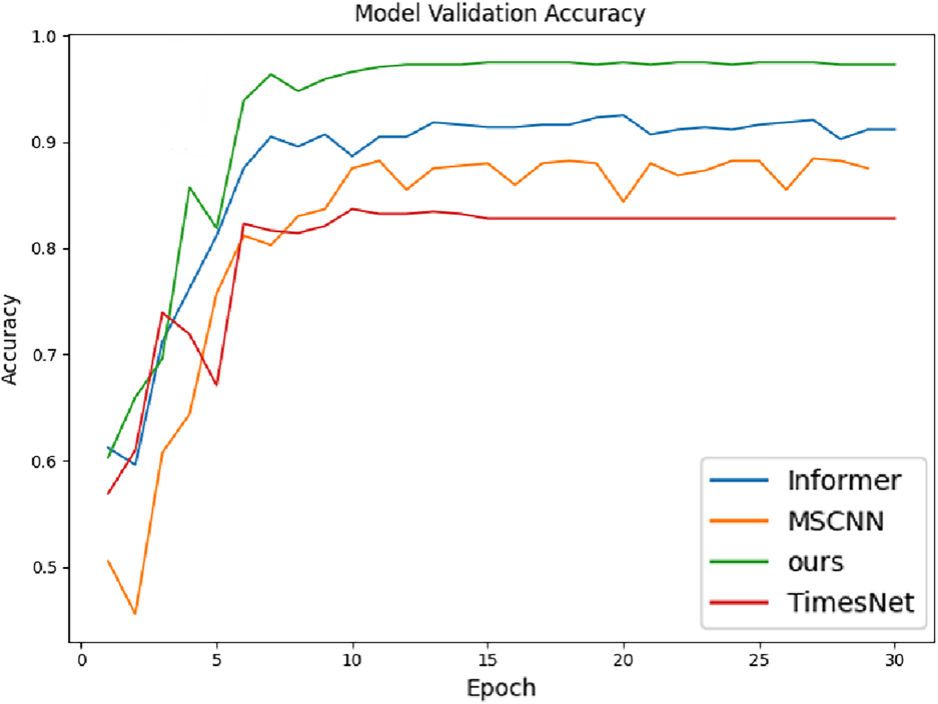
Accuracy changes of 30 iterations of four fault diagnosis methods in accumulator component diagnosis.

### Single fault diagnosis of different components

According to the three sampling rates of the experimental dataset, set the number of multi-scale channels of the time series feature extractor to 3. Table 2 summarizes the hyper-parameters of the proposed method in this experiment.

**Table 3:** Classification accuracy of fault diagnosis in different components.

Component		Classical methods		TCN	Transformers		Ours
		LDA	CNN	–	Informer	TimesNet	
Cooler	Accuracy	100%	100%	100%	100%	100%	**100%**
	Precision	100%	100%	100%	100%	100%	**100%**
	Recall	100%	100%	100%	100%	100%	**100%**
	F1 (weighted)	100%	100%	100%	100%	100%	**100%**
Valve	Accuracy	93.88%	94.78%	46.71%	95.92%	95.69%	**100%**
	Precision	93.86%	95.05%	21.82%	95.94%	95.77%	**100%**
	Recall	93.88%	94.78%	46.71%	95.92%	95.69%	**100%**
	F1 (weighted)	93.70%	94.55%	29.75%	95.83%	95.56%	**100%**
Pump	Accuracy	98.64%	97.27%	57.60%	99.77%	**100%**	99.77%
	Precision	98.72%	97.39%	56.47%	99.78%	**100%**	98.42%
	Recall	98.64%	97.27%	57.60%	99.77%	**100%**	98.42%
	F1 (weighted)	98.64%	97.27%	47.42%	99.77%	**100%**	98.41%
Acc.	Accuracy	68.71%	72.78%	55.78%	91.38%	83.67%	**97.27%**
	Precision	68.30%	71.27%	47.18%	91.47%	84.23%	**97.06%**
	Recall	68.71%	72.79%	55.78%	91.38%	83.67%	**97.05%**
	F1 (weighted)	68.60%	71.40%	48.81%	91.39%	83.58%	**97.03%**

Bold indicates the best value for the indicator in that row.

To verify the effectiveness of the proposed method, several methods are introduced for comparison. First, compare with classical fault diagnosis methods, namely Linear Discriminant Analysis (LDA)^7^, 1D Convolutional Neural Network (1D-CNN), and Multi-channel 1D Convolutional Network^29^; Secondly, compare with the Time Convolutional Network (TCN) in time series methods^30^; Then, compare with the cutting-edge Transformer model constructed time series method. The experimental results are recorded in Table 3, and the changes in training loss and validation accuracy during the training process are shown in Figs. 5 and 6, respectively. The model’s convergence speed during the training process is significantly higher than the other models.

**Figure 7:**
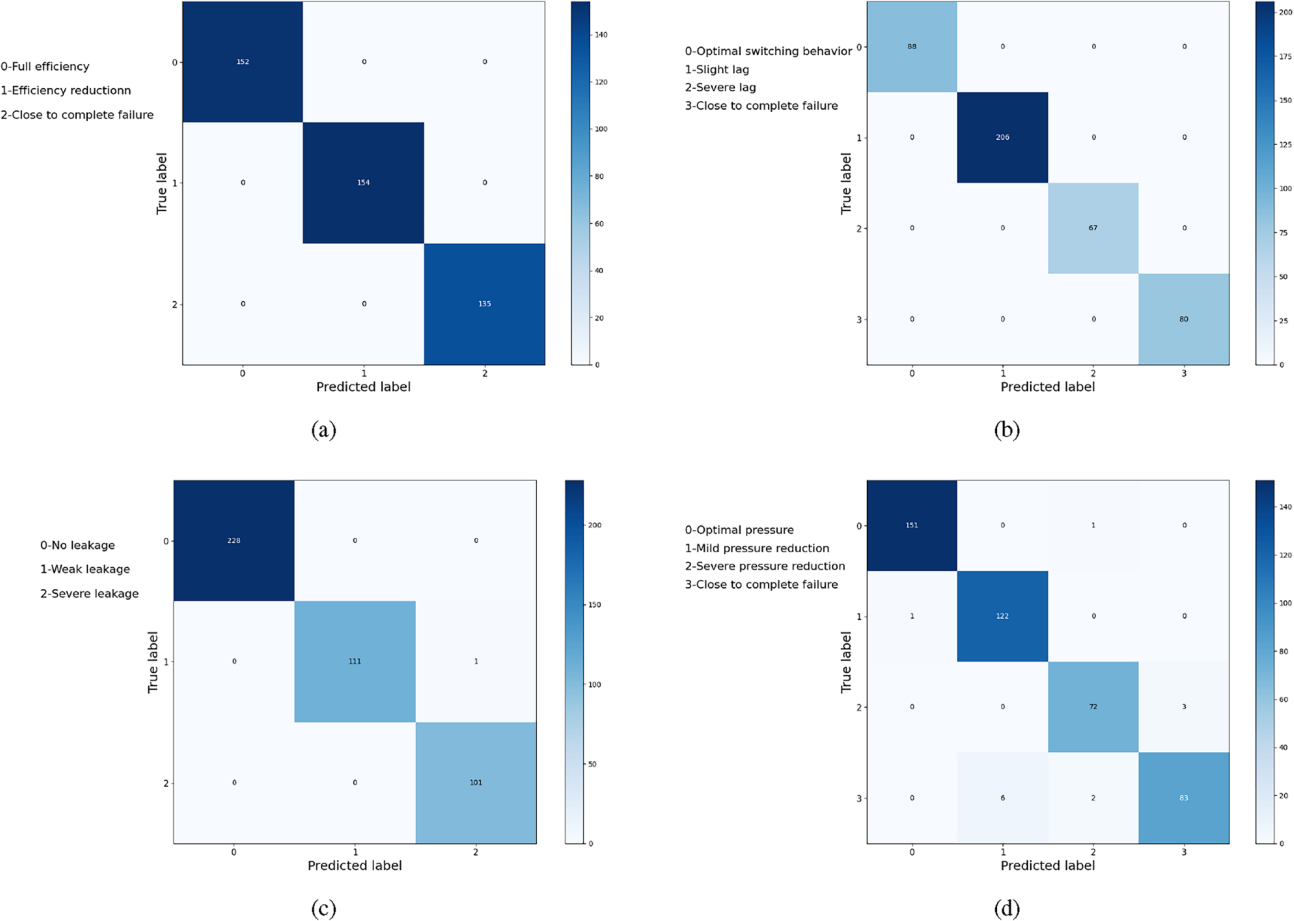
Confusion matrix under different components: (**a**) cooler, (**b**) valve, (**c**) pump, (**d**) accumulator.

In order to display the results more intuitively and provide a misclassification analysis, the confusion matrix for the detection of four types of individual components is shown in Fig. 7. The vertical axis represents the true labels of the samples, while the horizontal axis represents the predicted labels of the samples. In the fault diagnosis of the cooler and hydraulic valve, all samples are classified into their respective categories. Although in the fault detection of the main pump and accumulator, only a few samples are misclassified into adjacent categories. The described misclassifications reveal that the similarity of sensor data between continuously degrading states may lead to confusion in fault recognition^31^.

### Performance under noise environment

To better match real industrial control scenarios and test the model’s resistance to noise, and to verify the proposed method’s ability to suppress noise, Gaussian white noise with different signal-to-noise ratios is added to the selected fault signals. The signal-to-noise ratio is calculated as follows:

13 $$\begin{aligned} \begin{array}{c} \textrm{SNR}=10\cdot \lg \left( \frac{P_s}{P_n} \right) \end{array} \end{aligned}$$

In the equation, $P_s$ represents the power of the signal, and $P_n$ represents the power of the added noise.

**Figure 8:**
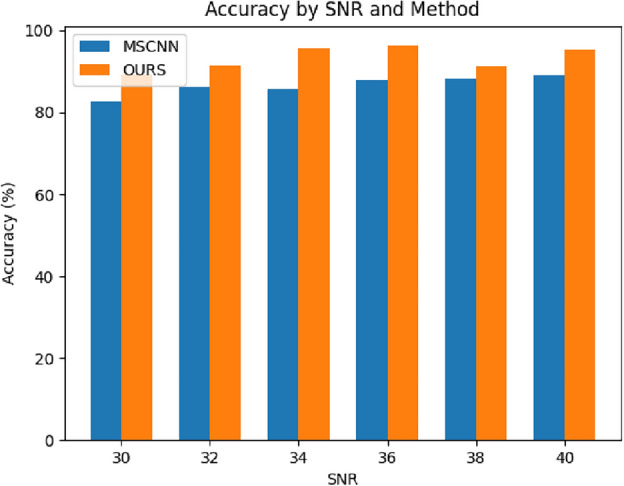
Accuracy of hydraulic accumulator fault diagnosis under different SNR.

As shown in Fig. 8, in the experiment, the SNR is set to increase from 30 to 40, which means the power of the noise is set to increase from 0.1 to 1% of the signal power. Generally speaking, the accuracy of fault detection increases with the increase of signal-to-noise ratio, and the noise environment has different effects on the diagnosis results of different components. In the fault detection of the cooler and valve, the influence of noise on the results is very small, and the diagnostic accuracy is close to 100.0%. However, in the fault diagnosis of the pump and accumulator with more complex fault scenarios, the noise environment has a significant impact on the diagnostic accuracy. With the increase of signal-to-noise ratio, the accuracy of this method approaches to the accuracy in a noise-free environment. In hydraulic system fault diagnosis, the accuracy of this method only decreases slightly, indicating its robustness.

### Visualization analysis with t-SNE

To gain an intuitive understanding of the effectiveness of the proposed method, t-distributed stochastic neighbor embedding (t-SNE) was used in experiments to visualize the representations of test samples in a two-dimensional space. t-SNE is a non-linear dimensionality reduction technique, easy to optimize, and particularly suitable for visualizing high-dimensional datasets.

**Figure 9:**
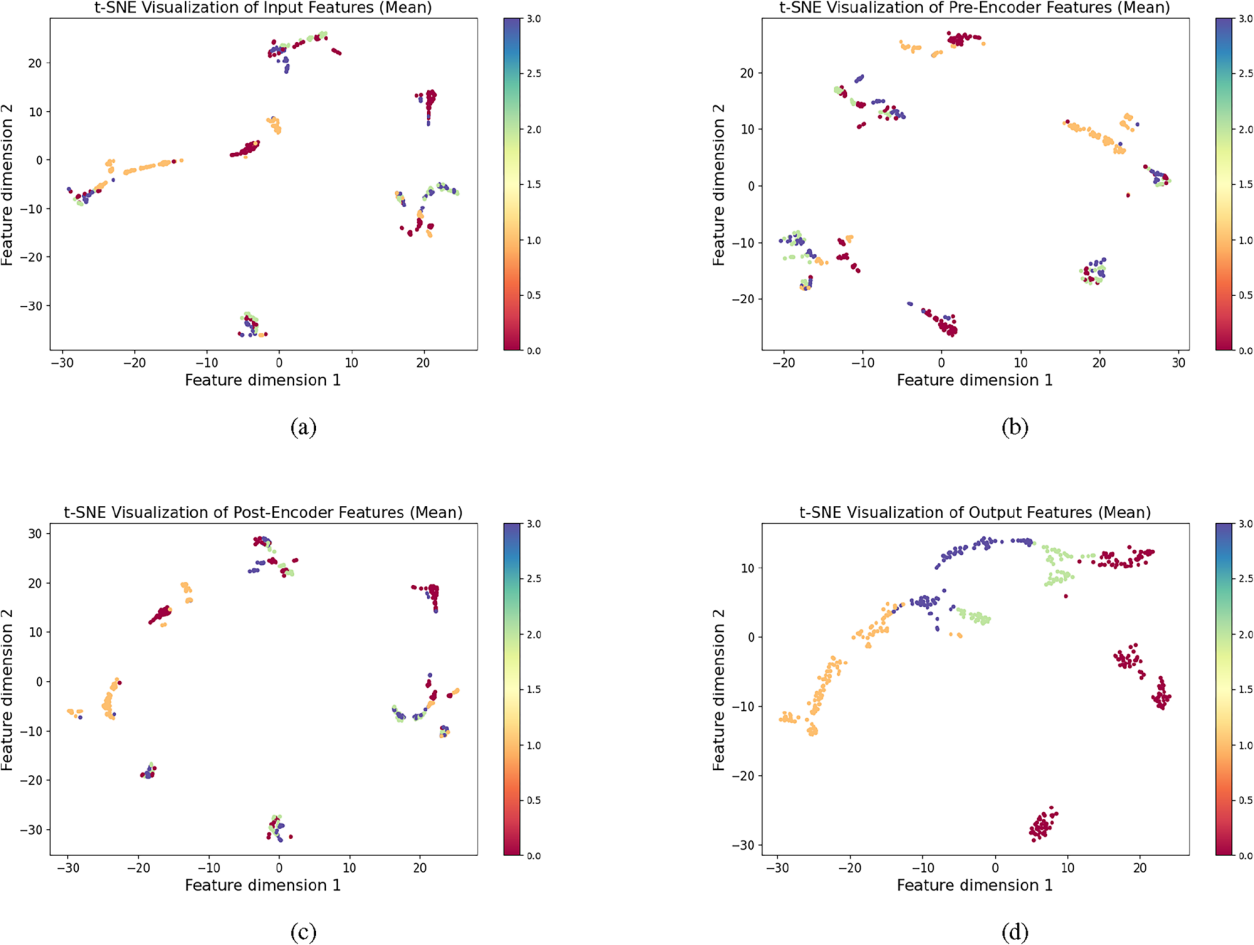
Use t-SNE to visualize the distribution changes of sample features: (**a**) Original signal, (**b**) Multi-scale feature extract signal, (**c**) Feature fusion signal, (**d**) Fault decoupled signal.

The accumulator is a critical energy storage component of the hydraulic system, and its failure may lead to the collapse of the entire system, with diagnostic difficulties being the most complex. In the experiment, visualization analysis of accumulator fault diagnosis was conducted. Firstly, test samples were fed into a trained model, and then features were extracted from the feature extractor and classifier. Subsequently, t-SNE was used to transform high-dimensional features into two-dimensional coordinates. Based on these coordinates, scatter plots were drawn and displayed in Fig. 9.

As shown in Fig. 9a, points from different categories of original data overlap with each other, indicating they cannot be simply separated by linear divisions. In contrast, features learned by the feature extractor exhibit better separability. Separation of data samples close to complete failure from samples of other categories indicates that the representations learned by the feature extractor facilitate preliminary distinction of fault categories. In the final Fig. 9d, data points from different categories are separated from each other, indicating that the method possesses strong non-linear mapping capabilities and can effectively identify faults.

## Results and discussion

This article proposes a new method for gear fault diagnosis based on a lightweight channel attention mechanism and transfer learning. The method addresses the problems of feature extraction, feature fusion, and fault decoupling for multi-sample rate sensor time series data and solves the issues of hidden and compound faults in hydraulic systems. The hydraulic system dataset is used to validate the classification and generalization ability of the proposed model. The conclusions are as follows:

1. This method has been proven to achieve accuracies of 100%, 100%, 100%, 99.77%, and 97.27% on the cooler C1, hydraulic valve V10, main hydraulic pump MP1, and accumulator A1–A4, respectively. Compared to classical methods and TCN, this method has absolute advantages in diagnosing the main hydraulic pump MP1 and accumulator A1–A4 datasets. It can extract more detailed features and effectively perform fault diagnosis. Compared to the latest Transformers time series models, it still has an advantage of around 5–10%.
2. Noise experiments show that this method has optimal diagnostic performance and generalization ability for different hydraulic components. It can achieve fast and accurate classification of multi-component faults in hydraulic systems. This is of great significance for solving compound fault problems in practical engineering applications.

In industrial environments, the differences in operating conditions and the issue of sample imbalance are more prominent. Future work will further explore the following aspects: (1) Expand the application scope, enhance the model’s generalization ability, and attempt to achieve condition adaptation through transfer learning methods. (2) Further research is needed to improve models in unlabeled industrial datasets, realizing semi-supervised or unsupervised learning while maintaining high accuracy.

### Supplementary Information

Supplementary Information 1.

Supplementary Information 2.

Supplementary Information 3.

## Data Availability

The fault diagnosis experimental data is sourced from an open dataset. The data that support the findings of this study are available from [doi: 10.1109/I2MTC.2015.7151267]. The datasets analyzed during the current study are available in the Kaggle repository, [https://www.kaggle.com/datasets/jjacostupa/condition-monitoring-of-hydraulic-systems/data].
