# Supplementary figures and images for: Hydraulic system fault diagnosis decoupling method based on 2D time-series modeling and self-attention fusion
Source: Sci Rep. 2024 Jul 7;14:15620. doi: 10.1038/s41598-024-66541-9 (PMC11228015; doi:10.1038/s41598-024-66541-9)

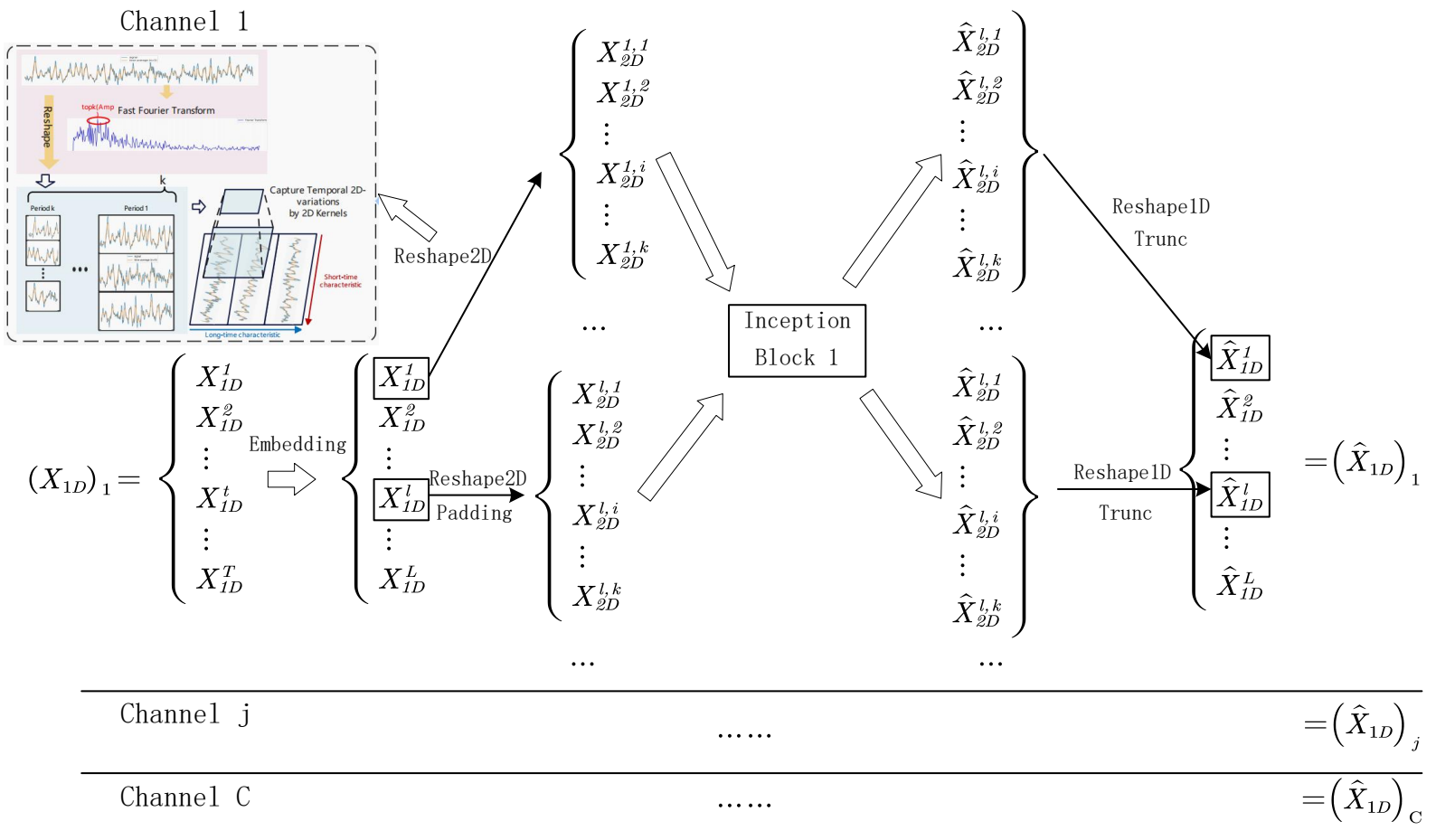

## Feature Fusion

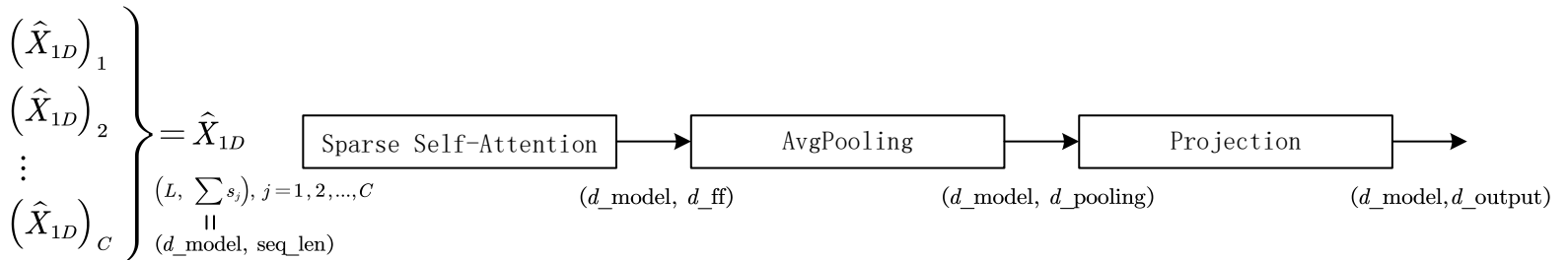

Supplement: Supplementary file 1 — Supplementary Information 1. [file 41598_2024_66541_MOESM1_ESM.pdf]
