# Supplementary material for: Hydraulic system fault diagnosis decoupling method based on 2D time-series modeling and self-attention fusion
Source: Sci Rep. 2024 Jul 7;14:15620. doi: 10.1038/s41598-024-66541-9 (PMC11228015; doi:10.1038/s41598-024-66541-9)
Supplement: Supplementary file 2 — Supplementary Information 2. [file 41598_2024_66541_MOESM2_ESM.pdf]

Table A2. Sensor Data Collected at 10Hz Showing Pearson Correlation.

Sensor	TS1	TS2	TS3	TS4	VS	CE	CP	SE
TS1	1	**0.99918917**	**0.999335682**	**0.998950279**	0.678712615	**-0.945480372**	**-0.908669586**	-0.1406436
TS2	**0.99918917**	1	**0.99882906**	**0.998139903**	0.67220596	**-0.945717569**	**-0.905810135**	-0.135829008
TS3	**0.999335682**	**0.99882906**	1	**0.998315064**	0.685129719	**-0.940870147**	-0.899736962	-0.149874098
TS4	**0.998950279**	**0.998139903**	**0.998315064**	1	0.682794469	**-0.955705284**	**-0.923454472**	-0.145083389
VS	0.678712615	0.67220596	0.685129719	0.682794469	1	-0.636820193	-0.609701627	-0.524054205
CE	**-0.945480372**	**-0.945717569**	**-0.940870147**	**-0.955705284**	-0.636820193	1	**0.972298006**	0.100325555
CP	**-0.908669586**	**-0.905810135**	-0.899736962	**-0.923454472**	-0.609701627	**0.972298006**	1	0.102600136
SE	-0.1406436	-0.135829008	-0.149874098	-0.145083389	-0.524054205	0.100325555	0.102600136	1
