# Supplementary material for: Hydraulic system fault diagnosis decoupling method based on 2D time-series modeling and self-attention fusion
Source: Sci Rep. 2024 Jul 7;14:15620. doi: 10.1038/s41598-024-66541-9 (PMC11228015; doi:10.1038/s41598-024-66541-9)
Supplement: Supplementary file 3 — Supplementary Information 3. [file 41598_2024_66541_MOESM3_ESM.pdf]

Table A3. Sensor Data Collected at 1Hz Showing Pearson Correlation.

Sensor	FS1	FS2
PS1	1	-0.610981189
PS2	-0.610981189	1
